# Patient satisfaction, patient safety and increasing violence against healthcare professionals

**DOI:** 10.12669/pjms.311.6965

**Published:** 2015

**Authors:** Shaukat Ali Jawaid

Increasing commercialization and corruption coupled with wide spread unethical practices by the medical profession has lead to increased violence against the healthcare professionals which has highlighted the importance of patients satisfaction and patient safety. A study by Hongzing Yu et al ^1^ being published in this issue from China has also highlighted numerous cases of violence against healthcare professionals leading to death of some doctors as well as nurses by patient’s relatives. The reasons for this violence according to the authors are poor quality of services, increased awareness of patients about their rights and their willingness to knock the doors of courts to seek justice. They have further reported that more than one million cases of violence against healthcare professionals are reported every year in China.^2^ Negative media reports about hospitals and doctors, out of pocket medical expenditures by the patients and lack of trust in doctors and hospitals have been reported to be some of the causative factors.^3^^-^^5^ Medical malpractice claims in courts in China is also reported to have increased by 7.6% from 2009 to 2010.^2^

This situation seems to be similar in most of the countries in this region including India, Bangladesh and Pakistan. Ali Khawaja and Hira Irfan^6^ reported that medical professionals are among the highly vulnerable professionals who are being increasingly subjected to violence by the patients and their relatives. According to them almost 77% of physicians have faced either verbal or physical abuse in Pakistan.^7^ Another study from Pakistan by Nazish Imran et al^8^ reported that 74% of respondents in a retrospective exploratory cross sectional study in a public sector healthcare facility in Lahore were victims of violence during the preceding twelve months with verbal abuse being the most common. Those exposed to violence experienced high level of psychological distress. Emergency Departments were the most common place where violence was witnessed and the sources of violence were patient’s relatives along with negative media reports besides irritating staff attitude.

Madhok from India has reported doctors being molested, thrashed and abused by lay public for a trivial fault. According to them the causes of violence were lack of communication between doctor and the patient, poor image of medical profession, lack of faith in judicial system and the police, besides insufficient security for doctors.^9^

A report from Bangladesh also throws light on the fact that violence in healthcare sector has been increasing at an alarming level throughout the country. Doctors become an easy target for the blame game by sensational media reports. Since a wide gap exists between the patient’s expectations and the reality, patients who feel they have not been looked after properly then take the matters in their own hands. These incidents have certainly decreased the self esteem of the doctors.^10^ Another report suggests that almost 70% of doctors won’t want their own children to go into the medical profession.^11^

Violence against healthcare professionals has also been reported from Saudi Arabia. Algwaiz WM and colleagues in their questionnaire based study report that out of six hundred physicians and nurses, 383 (63.8%) completed the questionnaires at two public hospitals in Riyadh City during 2011. According to them more than two third (67.4%) respondents reported that they were victims of violence in the previous twelve months. Nurses were more likely to be exposed to violent incidents as compared to physicians. The reasons for this violent behaviour were reported to be excessive waiting time, shortage of staff and unmet patients demands. Verbal abuse was the most common and the assailants were mostly the patients relatives or friends followed by the patients themselves.^12^ A survey conducted among Physicians in Kuwait showed that 86% of doctors had experienced verbal insults or imminent threat of violence while 28% had experienced physical attacks of which 7% reported serious or fatal injury. Out of 781 violent incidents reported by the doctors, 73 involved physical attacks, eight involved physical assaults likely to have caused serious or fatal injury. ^13^

A national survey in Australia revealed that 58% of General Practitioners had experienced verbal abuse and 18% experienced property damage. GPs with fewer years of practice were more likely to experience verbal abuse as compared to those more experienced who becomes better equipped to deal with verbal abuse in such situations.^14^

 All this shows how wide spread this violence against the healthcare professionals has become and most reports blame the negative reporting of the media to be one of the important causative factors along with poor quality of service and unmet patients expectations from the healthcare facilities. The situation has become so bad that some even suggest that the healthcare professionals should learn Karate, Token do and other martial arts for self defense which will also boost their own confidence even if it is not used at all.^10^

While most of the triggers for violence or the causative factors are quite common, some may be peculiar to certain countries. For example in China, the authors report little incentives to the hospital directors to improve the quality of service, inefficient supervision besides problems in medical education like deficiencies in curriculum, absence of humanistic education, traditional teacher centered teaching methods, mushroom growth of medical schools which now number 644 which includes 146 institutions for higher education for medicine and 498 secondary vocational schools.( Chinese Education Statistical Year book 2012) This has resulted in six-fold increase in annual enrolment from 75,000 in 1998 to almost 450,000 in 2008.^15^ A similar situation is witnessed in countries like India, Pakistan and Bangladesh where there is mushroom growth of private medical and dental schools during the last couple of years with the result that the number of medical and dental institutions in private sector are much more as compared to the public sector in all these three countries. Many of these institutions do not have enough teaching and training facilities, their own affiliated teaching hospitals hence it has certainly affected the quality of training.

Medical profession and the healthcare facilities in Pakistan have so far refused any monitoring or accountability. Ideally if the medical profession and healthcare facilities could adopt some system of self monitoring wherein the professional specialty organizations can play a vital role, it will be better. However, it has not worked so far. The provincial government of Punjab established the Punjab Healthcare Commission (PHC) ^16^ for this purpose in 2010. Its main objective is to enhance patient satisfaction through redressal of complaints and ensure accountability at all levels. It has laid down minimum service delivery standards. PHC has now sole jurisdiction over cases pertaining to alleged medical negligence, malpractice or administrative failure & healthcare service providers have immunity against proceedings conducted against them at any other forum. Unfortunately the implementation of standards is erroneously perceived as a resource intensive process by service providers who are unaccustomed to any regulation so far. Punjab Healthcare Commission is also supposed to increase awareness amongst patients and healthcare establishments about their rights and responsibilities. However, instead of supporting the HEC, the medical profession has adopted a confrontational attitude towards it (PHC) which is not going to benefit them. If normal routine measures fail to yield any results and satisfy the patients, they might be forced to take law into their own hands as has been reported from China and that will be extremely painful. As such it is high time that authorities in these countries wake up and initiate some effective measures. Some of these could be as under:

1. Strict accreditation of medical and dental schools ordering closure of all those institutions with poor quality and standards. Those institutions which fail to get accreditation should be given some time to improve and make up the deficiencies failing which they should be closed down.

2. The number of enrollments in each medical and dental school should be based on the number, quality of teachers and other teaching facilities.

3. Doctors should never assure 100% cure and avoid negligence. 

4. Each patient should be adequately examined, investigated and treated.

5. Negligence should not be accepted under any circumstances.

6. Over confidence and too much cautiousness in patient care should also be avoided.

7. A realistic appraisal of the prevailing situation and communication to the patient and their attendants, relatives should be ensured. 

8. Patients should be involved in decision making regarding their treatment giving them adequate information about the possible complications.

9. Periodic updating of the condition of the patient to the attendants is necessary. Healthcare professionals in general and junior doctors in particular must develop proper communication skills and empathy.

10. Every healthcare facility must have a liaison office to deal with the media and respond to their queries. No media personnel the electronic media in particular should be allowed to enter the healthcare facilities without permission and they should be briefed by the liaison officer or the hospital spokesman. Media in particular has to show responsibility while reporting health issues and those covering health must have some core knowledge of health issues.

11. All healthcare facilities must have a proper boundary wall with adequate security system in place with monitored entry of public. Security guards should be posted inside the hospital particular in most sensitive areas like Emergency, Intensive Care Units, and Operation Theaters.

12. There is a need to improve the Doctor-Patient relationship. Under no circumstances the previous hospital or the referring doctor should be criticized. 

13. Avoid using words like you have come late.

14. In desperate situations, the patient must be given the choice of calling another doctor for second opinion if they so desire. 

15. Professional specialty organizations should play their role and come up with a mechanism of self monitoring of their members to ensure ethical medical practice.

16. Relationship between the Physicians and the Pharmaceutical Trade and Industry needs to be looked into and Guidelines prepared by the National Bioethics Committee on the subject should be implemented which will go a long way in eliminating unethical practices.

In short there is an urgent need to make the healthcare facilities a safe environment for the healthcare professionals to work only then they can be expected to work with devotion and dedication. Breaking News on the Television Channels regarding death of patients due to doctor’s negligence has only served to work against the patient’s own interest as now the healthcare professionals are very reluctant to handle serious cases, hence many precious lives which could have been saved are being lost. 
